# When Visual Richness Meets Cognitive Simplicity: How Interface Design Shapes Consumer Experience in Digital Fashion Commerce

**DOI:** 10.3390/bs16091675

**Published:** 2026-09-17

**Authors:** Xu Hu, Jing Zhang

**Affiliations:** Global Management Department, Kookmin University, Seoul 02707, Republic of Korea; houk_1119@kookmin.ac.kr

**Keywords:** digital consumer behavior, user interface design, interface complexity, dual-process theory, perceived shopping quality, online fashion shopping, digital commerce

## Abstract

This study examines how user interface type and interface complexity influence perceived shopping quality in digital fashion commerce. Drawing on dual-process theory and the online servicescape perspective, two 2 × 2 between-subjects experiments were conducted in apparel and cosmetics shopping contexts, with 192 valid participants in each study. The results show that peripheral-route-oriented user interfaces, characterized by visual richness and interactivity, consistently produce higher perceived shopping quality than central-route-oriented user interfaces. Low-complexity interfaces are also evaluated more favorably than high-complexity interfaces. However, the conditional effects differ across product categories. Study 1 provided qualified evidence that shopping frequency conditioned the joint effect of interface type and complexity, whereas in Study 2, interface type and complexity mainly showed direct effects. These findings suggest that effective digital shopping interfaces should combine visual richness with structural simplicity, while also accounting for differences across product categories. The study applies a dual-process-informed perspective to digital interface design and identifies product category and shopping engagement as potential boundary conditions in consumer responses to online shopping environments.

## 1. Introduction

The internet has now become the primary medium for most people worldwide to access information and consume content. As more consumers turn to online platforms for fashion purchases, this shift has significantly disrupted traditional brick-and-mortar retail. Online shopping has triggered a transformative economic shift, growing rapidly in the digital era (Manganelli & Nicita, 2022). Marketers are now leveraging internet-based technologies to attract consumers and achieve strategic goals (Boufim & Barka, 2021).

Contemporary consumers, influenced by growing global brand awareness, actively adopt new shopping technologies. Advances in digital technology have significantly reduced physical barriers, such as space and distance (Boufim & Barka, 2021). The rollout of fifth-generation (5G) technology has further enhanced consumers’ trust in digital services and created new opportunities, especially in online fashion retail (Mishra, 2023). Online retail has become essential for fashion retailers competing in the digital era. It has reshaped how consumers interact with the fashion industry by providing location-independent access to a diverse range of products and enabling seamless browsing and purchasing through smartphones. By integrating efficient search functions into their platforms, online retailers can cater to time-sensitive consumers and charge premium prices (Backus et al., 2022; Wohllebe et al., 2020).

The rapid expansion of digital commerce has fundamentally transformed how consumers interact with retail environments, shifting decision-making processes from physical stores to highly mediated online interfaces. In this context, user interface (UI) design has emerged as a critical strategic asset, shaping not only functional usability but also consumers’ cognitive and affective responses during online shopping experiences. Contemporary research emphasizes that digital interfaces are no longer passive channels of information delivery; rather, they actively construct consumption experiences and influence behavioral outcomes such as engagement, satisfaction, and loyalty (Garczarek-Bak et al., 2024). As service delivery moves from physical to digital spaces (Tran & Strutton, 2020), the concept of the online servicescape—encompassing user interface (UI) layout, content quality, navigation, and user experience—has become central to service design (Harris & Goode, 2010). Many businesses now apply design thinking to integrate innovation and encourage user adoption.

An increasing number of studies have pointed out the experience-centered nature of online platforms. In fashion e-commerce, researchers consistently find that simple and clean interfaces yield strong user outcomes. This design philosophy emphasizes intuitive functionality, precise layouts, and clear navigation, which together create enjoyable and frictionless shopping experiences (Y. Li & Fu, 2022).

To understand how consumers process online information and interact with interfaces, dual-process theory offers a valuable conceptual framework (Cacioppo et al., 1986). According to this theory, consumers engage with information through a central route involving deliberate cognitive processing or a peripheral route, which relies on intuition and emotional cues. Design elements such as layout, imagery, and font size directly affect satisfaction. Some studies suggest that when platforms offer a rich electronic servicescape, users may even form emotional attachments. Peripheral route UIs, often visually immersive, enhance satisfaction by presenting information more vividly (Jeannot et al., 2022). Interactive designs increase user engagement, and compared to centrally routed UIs that rely on dense text and fewer visuals, interactive designs reduce information overload and aid quick decision-making. This model has proven effective across various sectors, such as education, government, and commerce (Jongmans et al., 2022; Ruf et al., 2022). These insights imply that UI complexity may be a significant factor affecting shopping quality beyond the dichotomy of central and peripheral processing routes.

Importantly, consumer knowledge may moderate their perception of UI complexity. Experienced shoppers better navigate complex designs, which may influence satisfaction levels (Jeannot et al., 2022). Consumer characteristics such as gender, age, and shopping frequency also shape these perceptions. For instance, female consumers, who are typically more familiar with cosmetics, may favor peripheral cues, while male users may prefer central route processing when evaluating less familiar products (Khan et al., 2017; Muthupriya, 2019).

Previous research confirms the significant influence of the online servicescape on consumer behavior. While online shopping is now widespread, interface design and cognitive processing still shape the user experience. Well-designed fashion platforms can evoke positive emotions, thereby enhancing engagement, loyalty, and word-of-mouth advocacy (Khare et al., 2023). Despite these advances, several critical gaps remain. First, prior research has predominantly examined UI design from either a cognitive perspective (e.g., usability, efficiency) or an affective perspective (e.g., aesthetics, enjoyment), with limited integration of these dual processes within a unified framework. Although dual-process perspectives have gained traction in consumer research (Zhang et al., 2024), their application to UI design in online shopping contexts remains underdeveloped. Second, while immersive and experience-oriented design has been widely acknowledged, the underlying psychological mechanisms through which UI design influences user experience quality remain insufficiently specified. Third, existing studies often focus on general e-commerce settings, with relatively limited attention to category-specific contexts such as online fashion platforms, where experiential processing is particularly salient.

The present study addresses this gap by investigating how UI type (central vs. peripheral) and UI complexity (high vs. low) affect perceived shopping quality in two fashion-related product categories: apparel and cosmetics. We also examine how user characteristics, including gender, age, purchase frequency, and years of online shopping, moderate these effects. By integrating dual-process theory with research on user interface design and the online servicescape, this study provides theoretical insights and practical guidance for fashion e-retailers seeking to enhance user immersion and optimize platform performance.

By doing so, this research contributes to the literature in three important ways. First, it advances current understanding of UI design by integrating dual-process theory into digital consumption contexts, offering a more nuanced explanation of how users process interface stimuli. Second, it enriches the UX literature by linking interface design configurations to perceived shopping quality through a dual-process-informed conceptual framework. Third, it provides actionable insights for practitioners seeking to design online shopping platforms that effectively balance functionality and experiential richness in increasingly competitive digital environments.

## 2. Theoretical Development

### 2.1. Dual-Process Theory and Interface Design Orientation

Consumers’ online purchasing behavior toward fashion apparel brands reflects the dual processing mechanisms of the central and peripheral routes (M. S. Rahman & Mannan, 2018). According to dual-process theory, consumers who prefer the central route engage in active, effortful cognitive processing. In contrast, those who rely on the peripheral route respond more to external contextual cues that shape their judgment (Cacioppo et al., 1986). The peripheral route operates through cues unrelated to the message’s core content, such as the attractiveness of the source, the number of arguments, or emotional appeals. In communication contexts, the peripheral route is particularly effective when the audience lacks high involvement or strong interest in the topic. Under these conditions, consumers with limited cognitive resources or motivation tend to rely on emotionally resonant and easily digestible cues, such as appealing visuals, authoritative figures, or engaging narratives (Lin et al., 2026).

Most studies on mobile applications have focused on textual and informational features, while few have explored the design of visual interfaces. Dual-process theory distinguishes between relatively effortful, analytic processing and more heuristic, cue-based processing. In digital shopping environments, however, processing routes are psychological responses of consumers rather than inherent properties of interface designs. Accordingly, the present research does not assume that a particular interface directly induces or constitutes a central or peripheral processing route. Instead, dual-process theory is used as a conceptual basis for distinguishing two interface design orientations that differ in the types of cues they make salient. Drawing from earlier research, we define a central-route-oriented user interface (C-UI) as an interface that foregrounds detailed textual information, functional explanations, and relatively limited visual stimulation. By contrast, a peripheral-route-oriented user interface (P-UI) foregrounds imagery, visual salience, simplified textual presentation, and interactive elements. These labels therefore describe theoretical design orientations rather than observed cognitive processing states. A well-designed interface can enhance the immersive shopping experience and improve its perceived quality. Based on this framework, we propose the following hypotheses:

**H1.**  *Consumers exposed to a peripheral-route-oriented user interface (P-UI) will report higher perceived shopping quality than consumers exposed to a central-route-oriented user interface (C-UI).*

### 2.2. UI Complexity and Processing Fluency

Some scholars have suggested that image-rich P-UI enhances immersion. However, users may find it easier to access relevant information when C-UIs incorporate an online service response strategy (Zuo et al., 2023). Other researchers have noted that complex C-UIs overloaded with text can cause cognitive fatigue and stress, reducing users’ attention span and response time and ultimately impairing their shopping experience (Zhou et al., 2022). These theoretical perspectives highlight UI complexity as a crucial factor influencing system design and usability. Generally, users prefer intuitive and easy-to-navigate interfaces that enhance browsing efficiency and facilitate seamless information retrieval. Studies have identified simplicity and clear operational flows as key factors in attracting consumer attention and enhancing purchase intentions (Schrepp et al., 2021).

Furthermore, prior research has linked UI complexity with perceived ease of use and perceived usefulness (Guo et al., 2022). Davis and Granić (2024) developed the Technology Acceptance Model (TAM) based on the Theory of Reasoned Action to explain and predict user acceptance of information technologies. TAM defines perceived ease of use as the extent to which users find an interface easy to operate, while perceived usefulness is their perception of how effectively the interface meets their functional needs. These two constructs continue to shape consumers’ willingness to adopt and engage with mobile commerce platforms (Nuralam et al., 2024). UI complexity directly affects the intuitiveness of the user experience and the learning curve required to master new interaction patterns (Bakaev et al., 2023). Designers must account for the user’s cognitive load and skill levels to enhance user satisfaction and ensure the long-term usability of the system.

In today’s digital age, UI design goes beyond visual appeal and functionality—it must adapt to users’ diverse needs. Complexity encompasses technical elements such as button size, font color, and layout, and the operational flow and cognitive demands required for comprehension. It determines whether users can intuitively recognize system functions and whether they are willing to invest effort in learning to use the platform effectively (Cena et al., 2022). Design teams must consider multiple factors, including users’ cognitive capacities, skills, and digital literacy. Overly complex interfaces increase user stress and require users to expend more energy navigating the system, often leading to reduced satisfaction (Zhou et al., 2022). Conversely, simple and intuitive designs enable smooth interaction without added learning costs, thereby enhancing the overall user experience. Adopting an inclusive design philosophy ensures users of varying abilities can operate comfortably within their cognitive comfort zones. This approach improves satisfaction and strengthens long-term usability and user retention.

UI complexity affects consumers’ perceived shopping quality. As complexity increases, so does the cognitive load required to process information (Bieniek et al., 2024), which can reduce users’ sense of immersion—a key characteristic of peripheral-route UIs (Ahmed et al., 2024). Likewise, extremely complex C-UIs can reduce perceived ease of use and perceived usefulness (Ahmed et al., 2024; Oberbichler et al., 2021), and disrupt users’ sense of challenge–skill compatibility (Tse et al., 2020). Based on this rationale, we propose the following hypothesis:

**H2.**  *UI complexity moderates the relationship between interface design orientation and perceived shopping quality, such that the advantage of P-UI over C-UI will be stronger under low-complexity conditions than under high-complexity conditions.*

### 2.3. Consumer Characteristics as Boundary Conditions

Consumer characteristics have a significant impact on consumer retention and repurchase intentions. Research suggests that habitual purchasing behaviors and prior experience contribute positively to customer quality, increasing the likelihood of repeat purchases (Bilovodska & Poretskova, 2024). Scholars have extensively explored how consumer knowledge shapes consumer attitudes and behaviors. Among the influencing factors, website complexity plays a key role, particularly for frequent shoppers who are more sensitive to interface usability and design (K. Makhitha et al., 2019). These findings underscore the importance for online retailers to prioritize consumer-friendly interfaces and develop targeted marketing strategies tailored to diverse consumer segments. In today’s digital consumer environment, demographic and behavioral traits, such as gender, age, purchase frequency, and online shopping history, shape individual consumer knowledge. These variations affect how consumers interact with online platforms and perceive the quality of shopping (Alrawad et al., 2023; X. Li et al., 2021; K. Makhitha et al., 2019).

#### 2.3.1. Demographic Characteristics

The rise of e-commerce has prompted growing scholarly interest in understanding gender differences in online shopping quality. Studies show that gender significantly influences consumers’ experiences on online fashion platforms (Muthupriya, 2019). Men and women exhibit different attitudes toward online shopping, engaging in distinct shopping behaviors and decision-making processes. Research indicates that male consumers typically approach shopping with rational, goal-oriented behaviors, whereas female consumers tend to prioritize emotional connections with products and personal preferences throughout their shopping journey (Kanwal et al., 2021). These differences underscore the importance of developing targeted, gender-specific marketing strategies to address the distinct purchasing needs of men and women. As technological innovations continue to reshape consumer behavior, marketers must closely monitor and respond to evolving gender-based patterns to keep their strategies relevant and competitive in today’s dynamic digital environment (Alrawad et al., 2023).

Despite the expanding research on gender and online shopping, significant challenges remain. For instance, K. M. Makhitha and Ngobeni (2021) found that although perceived online shopping complexity may vary by gender, their study did not support gender as a moderating variable in shaping online shopping attitudes. Their findings underscore the need for further research into the complex role of gender in digital shopping, particularly as online platforms continue to evolve.

Over the past two decades, online shopping has evolved dramatically, with demographic characteristics playing a critical role in shaping consumer purchasing behavior (Ma et al., 2022). Age, in particular, strongly influences how consumers interact with online shopping platforms, form preferences, and evaluate their overall shopping quality (Mammen et al., 2021). Researchers have found that mall attributes and perceived shopping value differ across gender and generational groups, emphasizing the need for retailers to consider these demographic factors when designing shopping environments. Prior studies have shown that most online grocery shoppers are under 55, with spending peaking among consumers in their 30s (Alrawad et al., 2023). These patterns suggest that while younger consumers frequently engage in online shopping, older consumers also make substantial contributions to total expenditures.

Different age groups demonstrate distinct preferences and behaviors in the online shopping context. Wu’s (2024) research reinforces this trend, identifying age as a key predictor of online shopping behavior and demonstrating that younger consumers tend to favor mobile-based platforms. Multiple factors influence how different age cohorts behave online, and perceived website complexity has emerged as a significant predictor of online shopping intentions. Age also moderates the impact of these perceptions. Therefore, retailers must understand the relationship between consumer age and UI complexity to develop targeted marketing strategies that resonate with specific demographic segments.

#### 2.3.2. Shopping-Experience Characteristics

Various factors, including internet accessibility and consumer behavior patterns, influence the frequency of online shopping (Mammen et al., 2021). Research has revealed an important phenomenon: consumers who frequently shop online tend to develop more diverse and extensive shopping knowledge. Their repeated exposure to e-commerce environments increases their familiarity with online platforms and sharpens their sensitivity to interface design. These experienced consumers can more easily detect subtle design elements such as navigation flows, product presentation styles, and payment options, allowing them to assess UI complexity more accurately (Shi et al., 2023).

In contrast, consumers who shop online less frequently often lack familiarity with a website’s features and functionalities. Without this knowledge, they may form overgeneralized perceptions of complexity, viewing the site as confusing or difficult to use. This cognitive bias can lead to confusion or frustration, which may discourage them from shopping online in the future (Jeannot et al., 2022). However, when a website features a simple and intuitive design, the influence of this bias tends to diminish. Even first-time users can navigate these platforms smoothly and enjoy a positive experience, quickly adapting to the interface.

In such cases, frequency of purchases becomes a crucial factor in shaping purchase intentions (Gonçalves et al., 2024). To enhance shopping satisfaction across different user segments, UI designers must account for varying levels of consumer knowledge and perceptions of UI complexity.

Consumers accumulate years of knowledge, which significantly shapes their attitudes, preferences, and overall satisfaction (Kettunen et al., 2020). As they gain more knowledge with e-commerce, they tend to develop greater trust in online platforms. Researchers have shown that prior knowledge significantly impacts consumer trust and repurchase intentions (X. Li et al., 2021), highlighting the importance of creating positive shopping experiences to cultivate customer loyalty. Studies have also found that perceived UI complexity influences consumers’ purchase intentions, particularly among those with limited consumer knowledge (Chaudhuri et al., 2021). These findings emphasize the need for e-retailers to reduce perceived complexity to enhance user experience and increase platform engagement (S. M. Rahman et al., 2022).

Over time, consumers often adapt to online shopping platforms by developing platform-specific preferences. These preferences help them navigate websites more efficiently and confidently, ultimately reducing their perception of complexity (Setiawan et al., 2020).

Taken together, existing research suggests that gender, age, purchase frequency, and years of online shopping shape perceptions of UI complexity, which in turn influence shopping quality (Mammen et al., 2021). However, researchers have yet to determine whether these moderating effects apply consistently across central- and peripheral-route-oriented UI types. Consumer characteristics were incorporated as potential boundary conditions. Rather than treating these indicators as a single latent construct, we examined four theoretically distinct characteristics separately: gender, age, shopping frequency, and years of online shopping. Gender and age capture demographic differences, whereas shopping frequency and years of online shopping represent behavioral engagement and accumulated experience with digital shopping environments. Based on this gap, we propose the following hypothesis:

**H3a.**  *Gender moderates the joint effect of interface design orientation and UI complexity on perceived shopping quality.*

**H3b.**  *Age moderates the joint effect of interface design orientation and UI complexity on perceived shopping quality.*

**H3c.**  *Shopping frequency moderates the joint effect of interface design orientation and UI complexity on perceived shopping quality.*

**H3d.**  *Years of online shopping moderate the joint effect of interface design orientation and UI complexity on perceived shopping quality.*

## 3. Methodology

**Figure 1:**
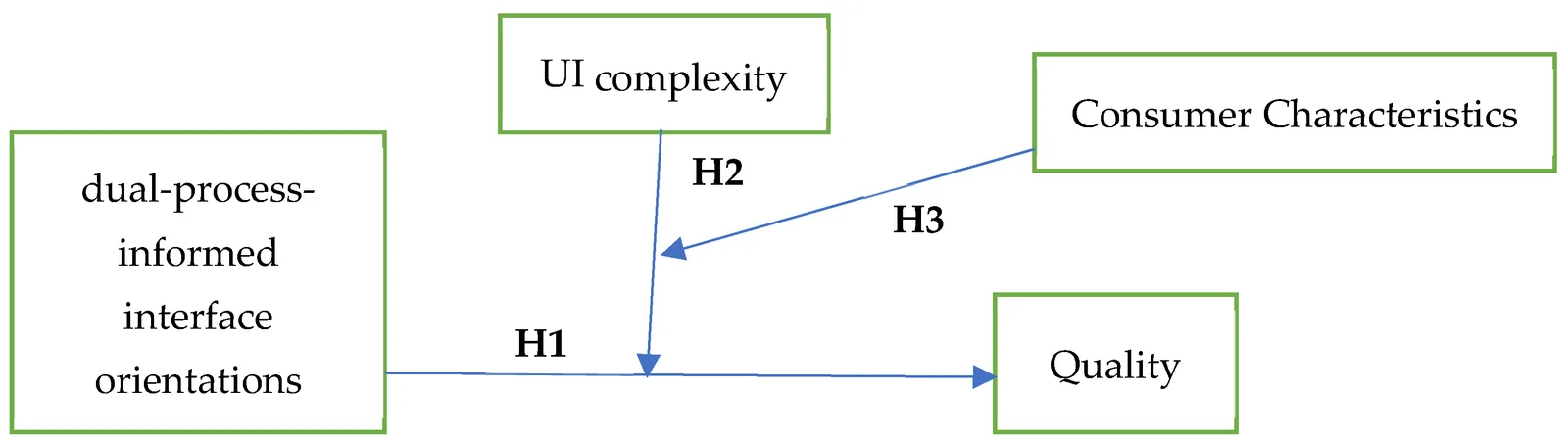
Conceptual Research Framework.

In this study, we designed one shopping application interface as a C-UI, which included detailed textual content, minimal imagery, and low interactivity. We developed another application as a P-UI, featuring simplified text, rich visuals, and high interactivity. We hypothesize that these different UI designs—based on dual-process-informed interface configurations—and their complexity levels may subtly influence consumers’ shopping performance and, in turn, their willingness to engage with the interface. In addition, we examine whether consumer characteristics moderate the relationship between perceived UI complexity and perceived shopping quality (see Figure 1).

### 3.1. Experimental Design

This study adopted a 2 (dual-process-informed interface orientations: C-UI vs. P-UI) × 2 (UI complexity: high vs. low) between-subjects factorial design. We conducted two experimental studies across different product categories—apparel and cosmetics—to explore the generalizability of the effects. We developed four interface types to represent each experimental condition: Group A (C-UI with low complexity), Group B (C-UI with high complexity), Group C (P-UI with low complexity) and Group D (P-UI with high complexity). We randomly assigned participants to one of the four experimental conditions and instructed them to complete product browsing and evaluation tasks on a simulated online fashion shopping platform. The study assessed how UI type and complexity level influence consumers’ perceived shopping quality and examined how individual consumer knowledge and characteristics moderate these relationships.

### 3.2. Variable Operationalization

This study operationalized four key constructs grounded in dual-process theory and prior research on online servicescapes and digital consumer behavior. First, UI type was treated as the primary independent variable and conceptualized according to the distinction between central-route and peripheral-route-oriented information processing. Central-route-oriented user interfaces (C-UIs) emphasized text-based content, functional explanations, and relatively limited visual stimulation, whereas peripheral-route-oriented user interfaces (P-UIs) incorporated image-oriented layouts, visually immersive elements, and higher levels of interactivity. This operationalization differentiates interface configurations according to the relative salience of informational versus visual and experiential design cues; it does not directly measure the cognitive processing route adopted by consumers when interacting with online shopping platforms.

Second, UI complexity was modeled as a moderating variable and manipulated through differences in navigation depth, information density, layout structure, and visual organization. High-complexity interfaces contained denser textual information, smaller icons, and less intuitive navigation structures, thereby increasing cognitive demands during browsing. In contrast, low-complexity interfaces adopted simplified layouts and clearer operational flows to enhance usability and reduce cognitive burden.

Perceived shopping quality served as the dependent variable and referred to consumers’ overall evaluations of the online shopping experience. Consistent with prior research on online servicescapes and customer experience, this construct captured users’ perceptions of platform professionalism, shopping satisfaction, and willingness to recommend the platform to others.

In addition, consumer characteristics were incorporated as moderating variables to examine whether individual differences influence perceptions of UI complexity and shopping quality. Following previous studies on online shopping behavior, these variables included gender, age, purchase frequency, and years of online shopping. These indicators were selected because they reflect consumers’ familiarity with digital shopping environments and their ability to process interface-related information.

### 3.3. Measurement Instruments

The multi-item perceptual measures were assessed using seven-point Likert scales ranging from 1 (“strongly disagree”) to 7 (“strongly agree”). Consumer characteristics were assessed using categorical demographic and behavioral measures. The measurement items were adapted from established studies in the fields of online consumer behavior, digital servicescapes, and technology acceptance, and were modified to fit the context of online fashion shopping platforms. The questionnaire consisted of four sections corresponding to dual-process-informed interface orientations, UI complexity, consumer characteristics, and perceived shopping quality.

Additional interface-evaluation items were adapted from Chen and Barnes (2007) to assess participants’ evaluations of information accuracy and platform responsiveness. Perceived ease of use was assessed using three items adapted from Chin et al. (2008) and was used as an indirect perceptual indicator related to the complexity manipulation.

Consumer characteristics were measured using demographic and behavioral indicators commonly adopted in online shopping research. Specifically, participants reported their years of online shopping, purchase frequency, gender, and age group. Years of online shopping were categorized into four levels: less than one year, 2–3 years, 4–5 years, and more than six years. Shopping frequency was dichotomously coded for the moderation analyses, with participants who shopped online once per week or less coded as 1 and those who shopped online twice or more per week coded as 0. Age was grouped into seven categories ranging from under 18 years old to over 60 years old.

Perceived shopping quality. We adapted items from “Phil” Klaus and Maklan (2012) and De Oliveira Santini et al. (2020), such as “The content on this platform is professional; they know what they are doing” and “I would recommend this platform to others.” The measurement items and coding schemes used in the reported analyses are provided in Appendix A, while the experimental conditions and stimulus design are summarized in Appendix B.

## 4. Study 1

In the fashion context, people view apparel as more than just clothing—it holds economic, social, and practical value in contemporary society. Clothing serves as a cultural symbol that drives economic activity and meets functional needs in daily life. Fashion apparel expresses individual style and aesthetic preferences while also reflecting broader societal values and lifestyles. As a result, apparel plays multiple roles: it meets personal consumer needs, enables the expression of identity, and drives the evolution of fashion trends (Akhilendra & Aravendan, 2023).

### 4.1. Study 1: Experimental Design and Procedure

**Figure 2:**
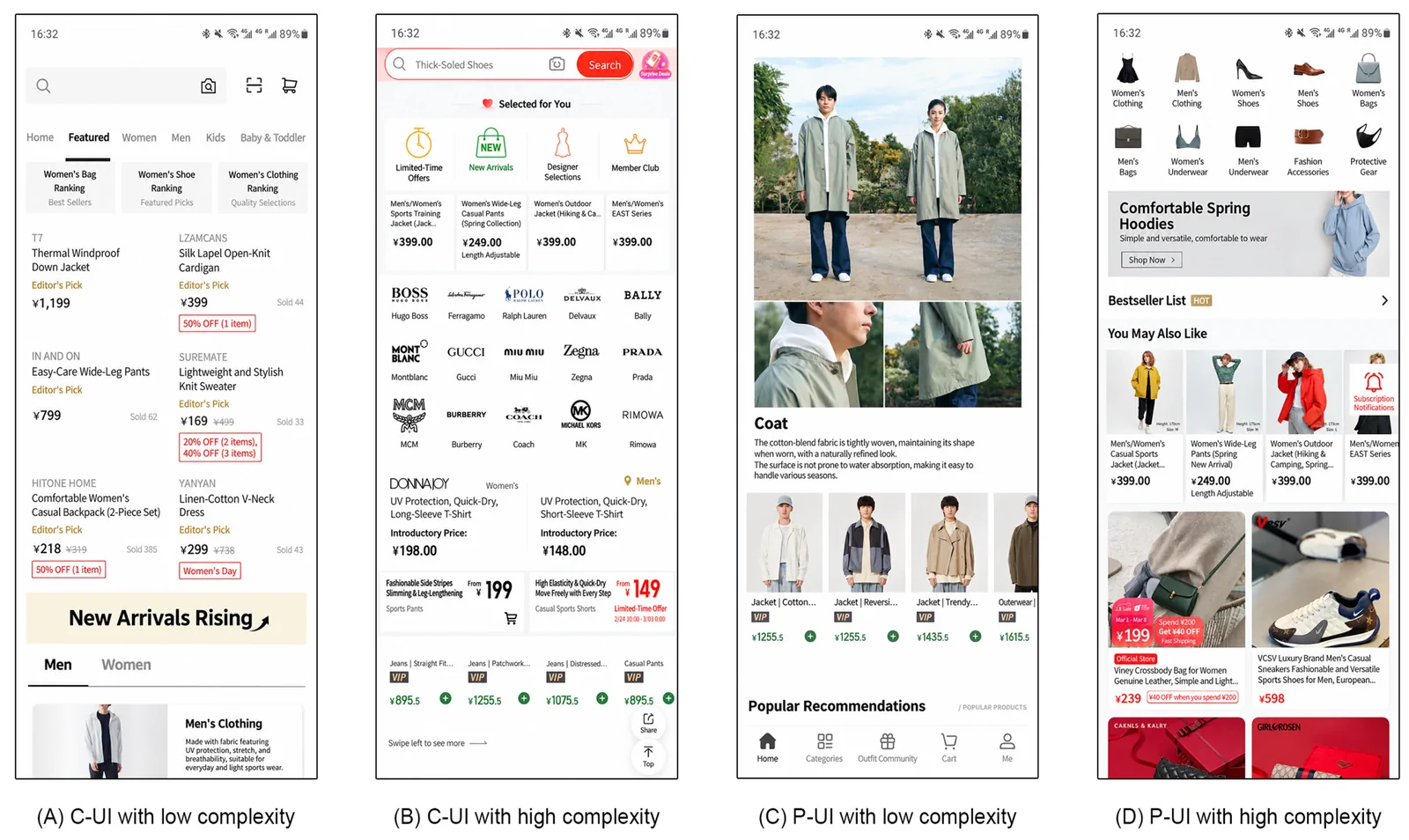
Stimuli of Online Apparel Shopping Platform.

To examine how UI type and complexity influence perceived shopping quality, we designed Study 1 as a 2 × 2 between-subjects factorial experiment. We developed four types of apparel shopping interfaces to represent each experimental condition and randomly assigned each participant to one of the four groups. We crafted the UI stimuli (see Figure 2) to reflect realistic online shopping scenarios and distributed them through the Credamo platform, which researchers widely use in Chinese online survey studies.

The required sample size was determined using G*Power (Version 3.1.9.6), a widely used tool for statistical power analysis in behavioral research. An a priori power analysis was conducted for a two-way ANOVA with four groups. Assuming a medium effect size (f = 0.250), a significance level of α = 0.050, and a desired statistical power of 0.800, the minimum required sample size was estimated to be 180 participants.

Based on this analysis, the target sample size for each study was set to exceed this threshold. After data cleaning, Study 1 retained 192 valid responses (48 per condition), exceeding the minimum sample size required to detect a medium-sized effect. The sample was approximately balanced by gender (50.5% male, 49.5% female).

### 4.2. Study 1: Results and Analysis

Perceptual Check of UI Complexity. Perceived ease of use (PEOU), assessed independently from perceived shopping quality, was used as an indirect perceptual check of the UI complexity manipulation. Participants in the low-complexity condition reported higher PEOU (M = 6.135, SD = 0.712) than those in the high-complexity condition (M = 5.931, SD = 0.965). However, this difference did not reach statistical significance, t(190) = 1.674, *p* = 0.096, 95% CI [−0.037, 0.446], Cohen’s d = 0.242. Thus, although the observed difference was directionally consistent with the intended manipulation, the perceptual check did not reach the conventional 0.05 significance threshold.

A dedicated perceptual manipulation-check measure of visual richness and experiential orientation was not included in the original questionnaire. Therefore, the distinction between C-UI and P-UI should be interpreted as an experimentally designed interface configuration rather than as a directly validated perceptual distinction.

To examine the effects of interface design orientation and UI complexity on perceived shopping quality in Study 1, a two-way ANOVA was conducted. As reported in Table 1, the analysis revealed a significant main effect of interface design orientation, F(1, 188) = 11.229, *p* = 0.001, ηp^2^ = 0.056. Participants exposed to the P-UI evaluated the shopping experience more positively than those exposed to the C-UI. Thus, H1 was supported in Study 1.

The analysis also revealed a significant main effect of UI complexity, F(1, 188) = 14.999, *p* < 0.001, ηp^2^ = 0.074, indicating that low-complexity interfaces generated more favorable evaluations of perceived shopping quality than high-complexity interfaces.

Importantly, the interaction between interface design orientation and UI complexity was not significant, F(1, 188) = 0.365, *p* = 0.546, ηp^2^ = 0.002. Thus, at the aggregate sample level, there was no evidence that the effect of interface design orientation on perceived shopping quality differed systematically between the high- and low-complexity conditions. H2 was therefore not supported by the factorial ANOVA. We subsequently examined whether this interaction varied as a function of individual consumer characteristics using moderated moderation analyses.

### 4.3. Study 1: Moderation Analysis

To test Hypotheses 3, PROCESS Model 3 was used to examine whether UI complexity (W) and consumer characteristics (Z) moderated the relationship between UI design orientation (X) and perceived shopping quality (Y).

As reported in Table 2, the three-way interactions involving gender, age, and years of online shopping were not statistically significant. Specifically, the interaction among interface design orientation, UI complexity, and gender did not reach the conventional 0.05 significance threshold, B = 1.050, SE = 0.555, t = 1.893, *p* = 0.060. Likewise, neither the interaction involving age, B = −0.302, SE = 0.616, t = −0.491, *p* = 0.624, nor that involving years of online shopping, B = −0.251, SE = 0.669, t = −0.375, *p* = 0.708, was statistically significant. Thus, H3a, H3b, and H3d were not supported.

In contrast, the three-way interaction among interface design orientation, UI complexity, and shopping frequency was statistically significant, B = 0.792, SE = 0.393, t = 2.016, *p* = 0.045, 95% CI [0.017, 1.567]. The addition of this three-way interaction accounted for a modest but statistically significant increment in explained variance, ΔR^2^ = 0.018, F(1, 184) = 4.063, *p* = 0.045. Conditional-effect analyses were therefore conducted to clarify the nature of this interaction.

**Figure 3:**
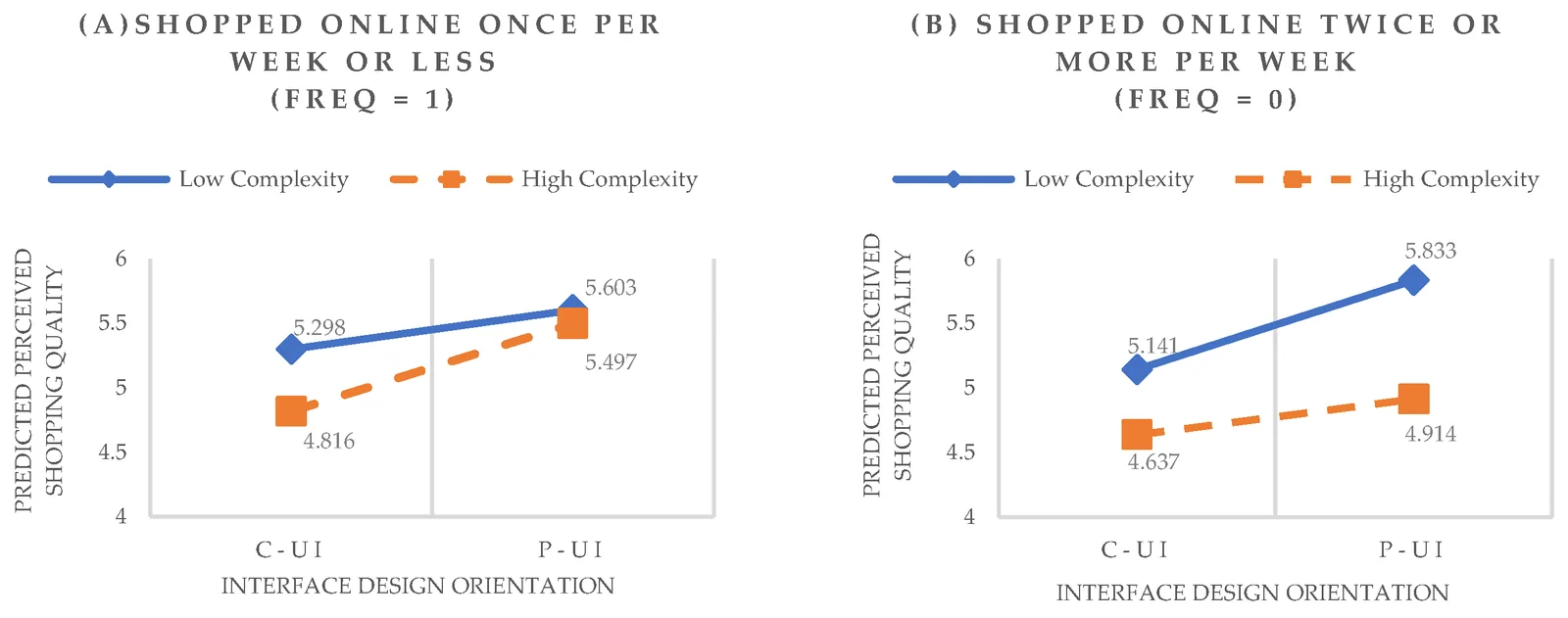
Conditional Effects of Interface Design Orientation and UI Complexity Across Shopping-Frequency Groups in Study 1. Note. Values represent model-predicted perceived shopping quality derived from PROCESS Model 3. Panel (**A**) represents consumers who shopped online once per week or less (freq = 1), whereas Panel (**B**) represents consumers who shopped online twice or more per week (freq = 0). C-UI = central-route-oriented user interface; P-UI = peripheral-route-oriented user interface.

Among consumers who shopped online once per week or less (freq = 1), P-UI produced significantly higher perceived shopping quality than C-UI under low complexity, B = 0.692, SE = 0.223, *p* = 0.002, 95% CI [0.253, 1.132], whereas the corresponding effect under high complexity was not statistically significant, B = 0.277, SE = 0.235, *p* = 0.239. Among consumers who shopped online twice or more per week (freq = 0), the pattern differed: the P-UI advantage was not statistically significant under low complexity, B = 0.305, SE = 0.294, *p* = 0.301, but was significant under high complexity, B = 0.682, SE = 0.278, *p* = 0.015, 95% CI [0.134, 1.230]. Figure 3 illustrates this conditional pattern.

However, the conditional interaction between interface design orientation and UI complexity was not statistically significant at either shopping-frequency level, F(1, 184) = 1.646, *p* = 0.201, or F(1, 184) = 0.867, *p* = 0.353. Accordingly, the significant three-way interaction should be interpreted as evidence that the pattern of the interface orientation × complexity relationship varied with shopping frequency, rather than as evidence of a statistically significant two-way interaction within either shopping-frequency group. Given the modest incremental variance explained by the three-way interaction (ΔR^2^ = 0.018), the result should be interpreted cautiously. Overall, H3c received qualified support, whereas H3a, H3b, and H3d were not supported.

### 4.4. Study 1: Implications

Study 1 offers several implications for understanding how UI design orientation and UI complexity shape perceived shopping quality on online apparel platforms. The findings show that P-UI design can improve consumers’ evaluations of the shopping experience, but its effect is not uniform across interface conditions or consumer groups. In particular, the moderated moderation results indicate that shopping frequency significantly qualifies the interaction between UI design orientation and UI complexity. By contrast, gender, age, and years of online shopping did not show statistically significant moderating effects. These results suggest that consumers’ current shopping behavior may be more relevant than broad demographic characteristics or accumulated online shopping experience in explaining how they respond to apparel-shopping interfaces.

Study 1 extends the application of dual-process theory to interface design by showing that distinctions commonly associated with central- and peripheral-route processing can inform theoretically differentiated UI design orientations. C-UIs foreground textual and functional information, whereas P-UIs rely more heavily on visual cues and interactive presentation. The results indicate that consumers’ evaluations are shaped not only by the amount or type of product information provided but also by how that information is visually organized and structurally delivered. The findings also point to UI complexity as an important condition under which interface effects may change. When complexity increases cognitive load or reduces processing fluency, it may weaken the advantage of visually rich interface design.

From a managerial perspective, online apparel retailers should pursue a design strategy that is visually engaging but structurally simple. Platforms can benefit from high-quality product images, model displays, styling suggestions, short videos, and interactive product views, provided that these elements are presented within a clear and manageable structure. Retailers should avoid cluttered layouts, excessive navigation depth, and redundant information that may interrupt consumers’ decision-making process. The results also suggest that shopping frequency can serve as a useful basis for user segmentation. Frequent shoppers may be more comfortable with richer functions and more detailed browsing tools, whereas less frequent shoppers may require clearer guidance, simplified layouts, and more intuitive decision paths. Overall, Study 1 shows that effective apparel interface design depends on balancing visual engagement, information clarity, and cognitive fluency.

## 5. Study 2

Cosmetic products are playing an increasingly important role within the fashion industry, with many fashion brands now treating their beauty lines as significant profit drivers (Kaswengi et al., 2020). Study 1 provided support for the main effect of interface design orientation and qualified evidence for a shopping-frequency boundary condition in the apparel context. Study 2 was designed to examine whether these patterns generalized to cosmetics.

### 5.1. Study 2: Experimental Design and Procedure

**Figure 4:**
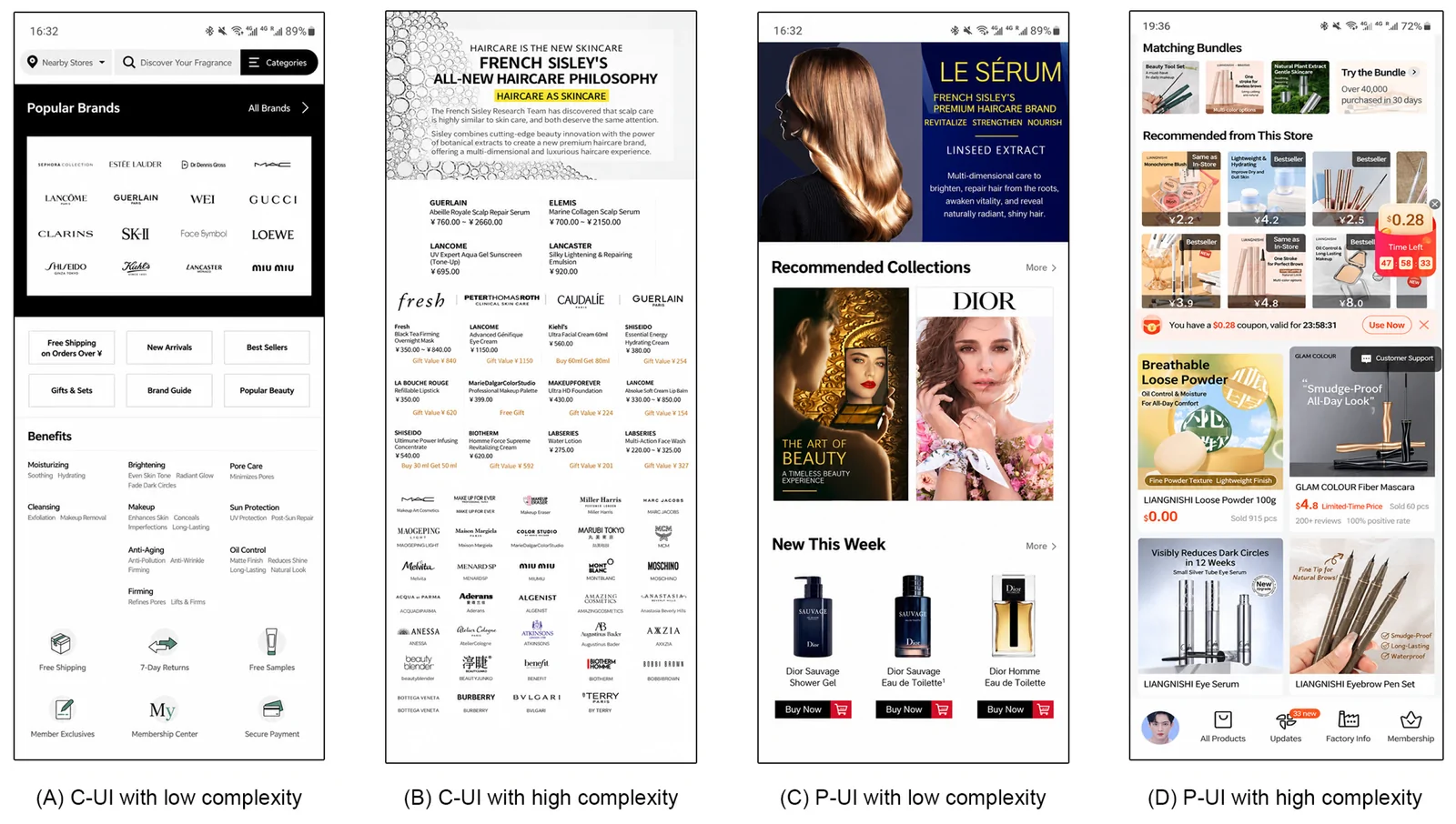
Stimuli of Online Cosmetic Shopping Platform.

Study 2 employed the same 2 (C-UI/P-UI) × 2 (high/low complexity) between-subjects factorial design, based on dual-process theory, as used in Study 1. However, we changed the product category to cosmetics to examine whether consumers would respond differently across product types (Figure 4). The experiment was conducted using the Credamo platform. Following the same sampling procedure as Study 1, a total of 192 valid responses were collected (48 per condition), ensuring sufficient statistical power for detecting medium-sized effects. The sample comprised an even split by gender, with 50% male and 50% female participants.

### 5.2. Study 2: Results and Analysis

Perceptual Check of UI Complexity. As in Study 1, PEOU was used as an indirect perceptual check of the UI complexity manipulation. Participants in the low-complexity condition reported significantly higher PEOU (M = 6.076, SD = 0.687) than those in the high-complexity condition (M = 5.795, SD = 0.995), Welch’s t(168.723) = 2.279, *p* = 0.024, 95% CI [0.038, 0.525], Cohen’s d = 0.329. This result was consistent with the intended complexity manipulation.

To examine the effects of interface design orientation and UI complexity on perceived shopping quality in the cosmetics-shopping context, a two-way ANOVA was conducted. As shown in Table 3, the analysis revealed a significant main effect of interface design orientation, F(1, 188) = 20.962, *p* < 0.001, ηp^2^ = 0.100. Participants exposed to the P-UI reported higher perceived shopping quality than those exposed to the C-UI. Thus, H1 was supported in Study 2.

The analysis also revealed a significant main effect of UI complexity, F(1, 188) = 5.630, *p* = 0.019, ηp^2^ = 0.029. Participants in the low-complexity condition evaluated the shopping experience more favorably than those in the high-complexity condition, indicating that reduced interface complexity was associated with higher perceived shopping quality.

However, the interaction between interface design orientation and UI complexity was not significant, F(1, 188) = 1.368, *p* = 0.244, ηp^2^ = 0.007. Thus, there was no evidence at the aggregate sample level that the advantage of P-UI over C-UI differed significantly between high- and low-complexity conditions. Accordingly, H2 was not supported by the factorial ANOVA in Study 2. We subsequently examined whether this relationship varied as a function of individual consumer characteristics using moderated moderation analyses.

### 5.3. Study 2: Moderation Analysis

To test H3 in Study 2, moderated moderation analyses were conducted using PROCESS Model 3. UI design orientation was entered as the independent variable, UI complexity as the first moderator, and consumer characteristics as the second moderator. As reported in Table 4, the interaction between UI design orientation and UI complexity was not significant in the models including gender, years of online shopping, or shopping frequency. In the age model, the interaction was also not statistically significant, B = 0.604, SE = 0.358, t = 1.690, *p* = 0.093, 95% CI [−0.101, 1.310].

The three-way interactions among UI design orientation, UI complexity, and consumer characteristics were also not significant. Specifically, the interactions involving gender, age, years of online shopping, and shopping frequency all failed to reach statistical significance. These results suggest that consumer characteristics did not meaningfully condition the moderating role of UI complexity in the cosmetics-shopping context. Accordingly, H3 was not supported in Study 2.

### 5.4. Study 2: Implications

Study 2 provides further evidence of how UI design orientation and UI complexity influence perceived shopping quality in the cosmetics-shopping context. The descriptive statistics and two-way ANOVA results show that P-UI design led to higher perceived shopping quality than C-UI design. Low-complexity interfaces were also evaluated more favorably than high-complexity interfaces. These findings suggest that, in cosmetics shopping, consumers respond positively to interfaces that are visually rich, interactive, and easy to process. Study 2 therefore confirms the general effectiveness of P-UI and further shows that interface simplicity remains important for improving perceived shopping quality.

Theoretically, Study 2 extends the dual-process perspective by showing that peripheral-route-oriented cues are effective in an experiential product category such as cosmetics. Cosmetics are closely associated with visual presentation, sensory imagination, appearance enhancement, and hedonic evaluation. In this context, consumers may rely more heavily on visual and affective cues when evaluating the shopping experience. P-UI can therefore improve perceived shopping quality by presenting products in a more vivid, intuitive, and engaging way. At the same time, the significant main effect of UI complexity suggests that consumers still value clarity and processing fluency, even in a visually driven product category.

However, the moderated moderation results show that H2 and H3 were not supported in Study 2. UI complexity did not significantly moderate the relationship between UI design orientation and perceived shopping quality, and consumer characteristics did not further condition this relationship. This finding suggests that, in the cosmetics context, UI design orientation and UI complexity function mainly as direct design factors rather than as interactive or conditional mechanisms. In other words, consumers’ evaluations of cosmetic-shopping interfaces appear to be less dependent on gender, age, shopping frequency, or years of online shopping.

This result also helps clarify the boundary conditions of the theoretical model. Compared with the apparel context in Study 1, the cosmetics context shows a more stable pattern of interface evaluation. Whereas Study 1 suggests that shopping frequency can shape how users respond to the combination of UI type and complexity, Study 2 indicates that such higher-order conditional effects are weaker in cosmetics shopping. One possible explanation is that cosmetics are more strongly tied to visual and experiential evaluation, leading consumers to respond more consistently to P-UI and low-complexity design regardless of individual characteristics.

From a managerial perspective, cosmetics e-retailers should prioritize visually engaging yet low-complexity interface design. Platforms can use high-quality product images, color displays, usage demonstrations, texture presentations, visual comparison tools, and interactive browsing features to enhance perceived shopping quality. These visual elements, however, should be organized within a clear and simple interface structure. Excessive layout density, complicated navigation, or redundant information may reduce processing fluency and weaken consumers’ evaluations of the platform.

Because consumer characteristics did not significantly moderate the effects in Study 2, cosmetics platforms may not need to rely heavily on demographic or experience-based segmentation when designing their basic interface structure. Instead, retailers should focus on creating a broadly accessible interface that combines visual appeal with simplicity and clarity. A standardized low-complexity P-UI may serve as an effective baseline design strategy for cosmetics-shopping platforms.

Overall, Study 2 suggests that an effective cosmetics-shopping interface should be visually rich, interactive, and easy to process. Unlike the apparel context, where UI effects depend more strongly on shopping frequency, the cosmetics context shows weaker conditional effects. Cosmetics e-retailers should therefore emphasize general interface quality, especially visual engagement and cognitive fluency, rather than designing highly differentiated interfaces based on consumer characteristics.

## 6. Discussion

### 6.1. Summary of Findings

This research examined how dual-process-informed interface orientations and UI complexity influence consumers’ perceived shopping quality on online fashion shopping platforms. Drawing on dual-process theory and the online servicescape perspective, the study treats UI design as a digital stimulus that shapes consumers’ cognitive and affective evaluations during online shopping. Across two experimental studies, central-route-oriented user interfaces (C-UIs), which emphasize textual and functional information, were compared with peripheral-route-oriented user interface (P-UIs), which emphasize visual richness, imagery, and interactive presentation.

The findings consistently support the positive role of P-UI across both product categories. In both the apparel and cosmetics contexts, participants evaluated P-UIs more favorably than C-UIs. This suggests that visually rich and interactive interface design is particularly important in online fashion shopping, where consumers often rely on visual cues, aesthetic judgment, and experiential impressions when evaluating a platform. These results support the argument that peripheral-route-oriented interface cues can enhance perceived shopping quality by making the shopping experience more vivid, intuitive, and engaging.

The findings also show that UI complexity plays a meaningful but context-dependent role. In both studies, low-complexity interfaces received more favorable evaluations than high-complexity interfaces, indicating that consumers generally prefer interfaces that are easier to process and navigate. However, the moderation results differed across the two product categories. In Study 1, conducted in the apparel context, UI complexity interacted with UI design orientation under certain consumer conditions. Study 1 provided qualified evidence that shopping frequency conditioned the relationships among UI design orientation, UI complexity, and perceived shopping quality. This suggests that consumers’ current shopping behavior plays an important role in shaping how they respond to different interface structures.

In contrast, Study 2, conducted in the cosmetics context, showed no significant higher-order interaction among UI design orientation, UI complexity, and consumer characteristics. Although P-UI and low-complexity design were still associated with higher perceived shopping quality, their effects operated mainly as direct design effects rather than conditional effects. In other words, consumer characteristics such as gender, age, shopping frequency, and years of online shopping did not significantly change how consumers responded to UI complexity in the cosmetics context.

Taken together, these findings suggest that the effectiveness of UI design on online fashion shopping platforms is both robust and context-sensitive. P-UI appears to be broadly effective across fashion-related categories, although the main effect of UI complexity was replicated across both studies, higher-order conditional patterns involving shopping frequency differed across product categories. Apparel shopping appears to involve a more conditional evaluation process, in which shopping frequency shapes how consumers interpret interface complexity. Cosmetics shopping, by contrast, shows a more stable response pattern, with consumers generally benefiting from visual richness and simplicity regardless of individual characteristics.

### 6.2. Theoretical Contributions

This research makes several theoretical contributions to the literature on dual-process theory, online servicescapes, and digital consumer behavior.

First, the study extends dual-process theory from message processing to interface design. Traditional dual-process theory distinguishes between central-route processing, which involves more deliberate and information-based evaluation, and peripheral-route processing, which relies more on contextual, affective, and heuristic cues. This study translates distinctions commonly associated with dual-process theory into two theoretically informed interface design orientations, without assuming that the interfaces themselves constitute or directly activate specific processing routes. C-UI represents a more information-oriented interface structure, whereas P-UI represents a more visually driven and experience-oriented interface structure. In this way, the research suggests that dual-process mechanisms are shaped not only by message content, but also by how digital environments organize, present, and visually frame information.

Second, this research contributes to the online servicescape literature by showing that UI design is not merely a functional component of online shopping platforms. Rather, it forms a central part of the digital consumption environment and directly shapes consumers’ perceived shopping quality. The findings suggest that consumers evaluate online fashion platforms through both informational and experiential cues. Visual layout, image richness, interactivity, navigation structure, and information density jointly influence whether consumers perceive a platform as professional, satisfying, and worth recommending. This supports a more integrated view of online servicescapes, in which interface design functions both as an information system and as an experiential environment.

Third, the study clarifies the role of UI complexity. Existing research often treats complexity as a negative design attribute because it increases cognitive load and reduces usability. The present findings offer a more nuanced interpretation. UI complexity can directly reduce perceived shopping quality, but its moderating role is not universal. In the apparel context, complexity becomes particularly important because consumers must evaluate multiple product-related cues, including style, fit, color, material, and self-presentation. The findings therefore position complexity primarily as a robust direct design factor, while Study 1 further suggests that higher-order conditional patterns involving complexity may emerge for particular consumer groups. In the cosmetics context, by contrast, complexity does not significantly condition the effect of UI type. This suggests that complexity should be understood as a context-dependent construct rather than as a uniformly negative interface feature.

Fourth, the findings identify shopping frequency as a potentially relevant behavioral boundary condition. In Study 1, shopping frequency significantly conditioned the joint effect of interface design orientation and UI complexity, whereas age and years of online shopping did not show comparable effects. However, this finding should be interpreted cautiously. Because the four consumer characteristics were examined in separate moderated-moderation models, multiple testing increases the risk of Type I error. Moreover, the three-way interaction accounted for a modest increment in explained variance (ΔR^2^ = 0.018) and did not replicate in Study 2. Accordingly, shopping frequency is better regarded as a potential category-dependent boundary condition that warrants further replication rather than as a general moderating mechanism.

Fifth, the study highlights product category as an important boundary condition in UI design theory. Apparel and cosmetics both belong to the broader fashion domain, but they involve different evaluation logics. Apparel shopping requires consumers to imagine fit, style coordination, material quality, and identity expression, making them more sensitive to the interaction between visual presentation and interface complexity. Cosmetics shopping, however, is more closely associated with sensory imagination, appearance enhancement, and hedonic evaluation. In this context, consumers may respond more consistently to visual and intuitive cues, which may reduce the role of higher-order interaction effects. By comparing these two categories, this study shows that UI design effects should not be assumed to operate uniformly across all fashion-related products.

Finally, this research makes a methodological contribution by demonstrating the value of examining higher-order interactions in digital consumer research. Main effects can show whether a particular interface type or complexity level improves perceived shopping quality, but they cannot explain when, why, or for whom these effects become stronger or weaker. The contrast between Study 1 and Study 2 shows that a fuller understanding of UI design effectiveness requires attention to product category, consumer behavior, and interaction effects. This approach provides a more refined explanation of how consumers evaluate online shopping platforms.

### 6.3. Managerial Implications

The findings offer several practical implications for fashion e-retailers, platform designers, and digital marketing managers.

First, online fashion platforms should give greater priority to P-UI design. Across both studies, P-UIs generated more favorable evaluations than C-UIs, suggesting that visually rich and interactive design is not merely decorative but directly contributes to consumers’ perceived shopping quality. Fashion retailers should therefore invest in high-quality product images, coherent visual layouts, interactive browsing features, model displays, styling suggestions, visual comparison tools, and immersive product presentations. These elements can help consumers form more positive impressions of the platform and enhance their overall shopping experience.

Second, platform designers should combine visual richness with interface simplicity. Although P-UI is generally beneficial, the findings also show that low-complexity interfaces are evaluated more favorably than high-complexity interfaces. Retailers should therefore avoid assuming that more design elements necessarily create a better experience. Excessive information density, crowded layouts, unclear navigation, and redundant interface modules may reduce processing fluency and weaken consumers’ evaluations. The most effective interface is not necessarily the most visually elaborate one, but the one that balances visual appeal with clear structure and intuitive use.

Third, apparel platforms should adopt a more segmented and adaptive design strategy. Study 1 shows that shopping frequency plays an important role in shaping consumers’ responses to UI design orientation and complexity. The qualified moderation pattern observed in Study 1 suggests that shopping frequency may provide a useful exploratory basis for interface segmentation. However, given the modest effect size and the absence of replication in Study 2, retailers should treat shopping frequency as a potential rather than definitive segmentation criterion.

Fourth, cosmetics platforms should focus on broadly accessible, low-complexity P-UI design. In Study 2, consumer characteristics did not significantly moderate the effects of UI type and complexity, suggesting that cosmetics-shopping interfaces may not require the same degree of segmentation based on demographic or experience-related variables. For cosmetics, a visually engaging, interactive, and easy-to-process interface may serve as an effective baseline strategy for a broad range of consumers. Cosmetics retailers should emphasize product imagery, color displays, texture presentation, usage demonstrations, and visual comparison tools while maintaining a clean and simple interface structure.

Fifth, retailers should adapt UI strategies to product category. Apparel platforms need to balance visual immersion with information clarity because consumers must evaluate fit, style, and self-presentation. Cosmetics platforms, by contrast, should emphasize vivid visual presentation and sensory imagination while still providing sufficient product information to support decision-making. A one-size-fits-all design approach is therefore unlikely to be optimal across fashion categories. Platform managers should consider product type, information requirements, and consumer familiarity when deciding how much visual richness and interface complexity to include.

### 6.4. Limitations and Future Research Directions

This research has several limitations that offer directions for future studies. First, the study examined only two fashion-related product categories: apparel and cosmetics. Although these categories are theoretically relevant and practically important, they do not represent the full range of online shopping contexts. Future research could extend the model to other categories, such as luxury goods, accessories, electronics, home products, groceries, or digital services. Such work would help determine whether the advantage of P-UI and the role of UI complexity apply beyond fashion-related products.

Second, the study used simulated online shopping interfaces and self-reported measures of perceived shopping quality. This design allowed for greater control over UI design orientation and complexity, but it may not fully reflect consumer behavior in real shopping environments. Future studies could use field experiments, clickstream data, eye-tracking, scrolling behavior, dwell time, conversion rates, or behavioral intention measures to examine how consumers actually interact with different interface designs. These methods would provide more direct evidence of attention allocation, cognitive load, and decision-making processes.

Third, the present study was limited in its direct validation of the psychological and perceptual mechanisms underlying the experimental conditions. The C-UI and P-UI conditions were designed as dual-process-informed interface configurations, but the study did not directly measure whether they activated central- or peripheral-route cognitive processing. In addition, perceived ease of use, measured independently from perceived shopping quality, provided only an indirect perceptual check of UI complexity. Although participants in the low-complexity condition reported higher perceived ease of use in both studies, the difference reached statistical significance only in Study 2. The original questionnaire also did not include dedicated measures of perceived visual richness, information orientation, or experiential interactivity to independently validate the C-UI and P-UI configurations. Accordingly, the findings should be interpreted as effects of theoretically designed interface configurations rather than as direct evidence of specific processing-route activation or of participants perceiving every manipulated attribute exactly as intended. Future research should incorporate direct measures of processing-route activation, cognitive load, processing fluency, visual attention, and affective engagement, together with independent manipulation checks of perceived complexity, visual richness, information orientation, and interactivity.

Fourth, consumer characteristics were operationalized through gender, age, shopping frequency, and years of online shopping. Although these variables are useful, they may not fully capture consumers’ product-specific knowledge or psychological tendencies. Future research could directly measure fashion involvement, cosmetics familiarity, product expertise, need for cognition, visual processing style, hedonic motivation, utilitarian motivation, technology readiness, and platform familiarity. These constructs may offer a more precise explanation of why consumers respond differently to UI complexity.

Fifth, the research was conducted with participants recruited through an online research platform under controlled experimental conditions. While this approach strengthens internal validity, the sample may not fully represent the diversity of global fashion consumers. Future studies could test the model across different cultural, regional, and market contexts. Cross-cultural research would be particularly valuable, as visual preferences, shopping habits, technology familiarity, and tolerance for interface complexity may vary across countries and consumer groups.

Finally, future research should examine how emerging technologies reshape the relationships among UI design, complexity, and perceived shopping quality. Online fashion platforms increasingly incorporate AI recommendation systems, virtual try-on tools, conversational agents, live commerce, augmented reality, and personalized interface layouts. These technologies may change how consumers experience complexity. A technically complex interface may feel simple if it provides personalized guidance, whereas a visually simple interface may feel insufficient if it lacks useful decision support. Future studies could therefore investigate how AI-enabled personalization and adaptive interface design interact with dual-process UI types to influence consumer experience.

Overall, this research shows that UI design affects perceived shopping quality through both general and context-dependent mechanisms. P-UI and low-complexity design generally improve consumers’ evaluations of online fashion shopping platforms. However, higher-order conditional patterns involving UI complexity may vary across product categories and levels of consumer engagement. Apparel shopping shows stronger conditional effects involving shopping frequency, whereas cosmetics shopping shows more stable direct effects of visual richness and simplicity. These findings offer a more nuanced understanding of UI design effectiveness and provide guidance for developing online fashion platforms that are visually engaging, cognitively fluent, and responsive to category-specific consumer needs.

## 7. Conclusions

This research demonstrates that interface design plays an important role in shaping consumers’ perceived shopping quality in digital fashion commerce. Across two experiments involving apparel and cosmetics, peripheral-route-oriented interfaces characterized by greater visual richness and experiential presentation consistently generated more favorable evaluations than central-route-oriented interfaces emphasizing textual and functional information. Low-complexity interfaces were also evaluated more positively across both product categories, highlighting the importance of cognitive simplicity in digital shopping environments.

At the same time, the findings demonstrate that interface effects are not entirely uniform across contexts. In apparel shopping, shopping frequency significantly conditioned the joint effect of interface design orientation and complexity, suggesting that consumers’ current behavioral engagement may influence their sensitivity to different interface configurations. In cosmetics shopping, by contrast, the effects of interface orientation and complexity were primarily direct, with no significant higher-order moderation by the examined consumer characteristics. These differences identify product category and shopping engagement as potentially important boundary conditions for understanding digital interface effectiveness.

The study therefore contributes to digital consumer behavior research by conceptualizing interface configurations through a dual-process-informed perspective while distinguishing interface design orientation from consumers’ actual cognitive processing routes. The findings suggest that effective digital fashion interfaces should not simply maximize visual stimulation or minimize information. Rather, successful design requires an appropriate balance between experiential richness and cognitive fluency. For practitioners, this implies that visually engaging interfaces should be supported by clear navigation, manageable information density, and category-appropriate interaction structures.

Nevertheless, because the present experiments manipulate bundles of interface characteristics and do not directly measure central or peripheral cognitive processing, the findings should be interpreted as evidence concerning dual-process-informed interface design orientations rather than direct evidence of processing-route activation. Future research incorporating direct measures of cognitive load, processing fluency, visual attention, and affective engagement could further clarify the psychological mechanisms through which interface design shapes consumer experience.

## Figures and Tables

**Table 1:** Two-way ANOVA Results for Perceived Shopping Quality in Apparel.

Effect	SS	df	MS	F	*p*	ηp^2^
types	12.403	1	12.403	11.229	0.001	0.056
cplx	16.568	1	16.568	14.999	0.000	0.074
types × cplx	0.403	1	0.403	0.365	0.546	0.002
Error	207.658	188	1.105	-	-	-

Note: The dependent variable was perceived shopping quality. Types refers to C-UI versus P-UI. Cplx refers to high versus low UI complexity. ηp^2^ = partial eta squared.

**Table 2:** Moderated Moderation Analysis of Perceived Shopping Quality in Apparel.

Moderator	Interaction Term	B	SE	t	*p*	95%CI	Conclusion
Gender	types × cplx × gender	1.050	0.555	1.893	0.060	[−0.045, 2.144]	Not Supp.
Age	types × cplx × age	−0.302	0.616	−0.491	0.624	[−1.518, 0.913]	Not Supp.
Years	types × cplx × years	−0.251	0.669	−0.375	0.708	[−1.571, 1.069]	Not Supp.
Freq	types × cplx × freq	0.792	0.393	2.016	0.045 *	[0.017, 1.567]	Supported

Note: * *p* < 0.05.

**Table 3:** Two-way ANOVA Results for Perceived Shopping Quality in Cosmetics.

Effect	SS	df	MS	F	*p*	ηp^2^
types	15.641	1	15.641	20.962	0.001	0.100
cplx	4.201	1	4.201	5.630	0.019	0.029
types × cplx	1.021	1	1.021	1.368	0.244	0.007
Error	140.277	188	0.746	-	-	-

Note: The dependent variable was perceived shopping quality. Types refers to C-UI versus P-UI. Cplx refers to high versus low UI complexity. ηp^2^ = partial eta squared.

**Table 4:** Moderated Moderation Analysis of Perceived Shopping Quality in Cosmetics.

Moderator	Interaction Term	B	SE	t	*p*	95%CI	Conclusion
Gender	types × cplx × gender	0.500	0.481	1.040	0.300	[−0.449, 1.449]	Not Supp.
Age	types × cplx × age	−0.596	0.500	−1.192	0.235	[−1.583, 0.391]	Not Supp.
Years	types × cplx × years	0.416	0.571	0.729	0.467	[−0.711, 1.544]	Not Supp.
Freq	types × cplx × freq	−0.563	0.548	−1.027	0.306	[−1.643, 0.518]	Not Supp.

**Figure A1:**
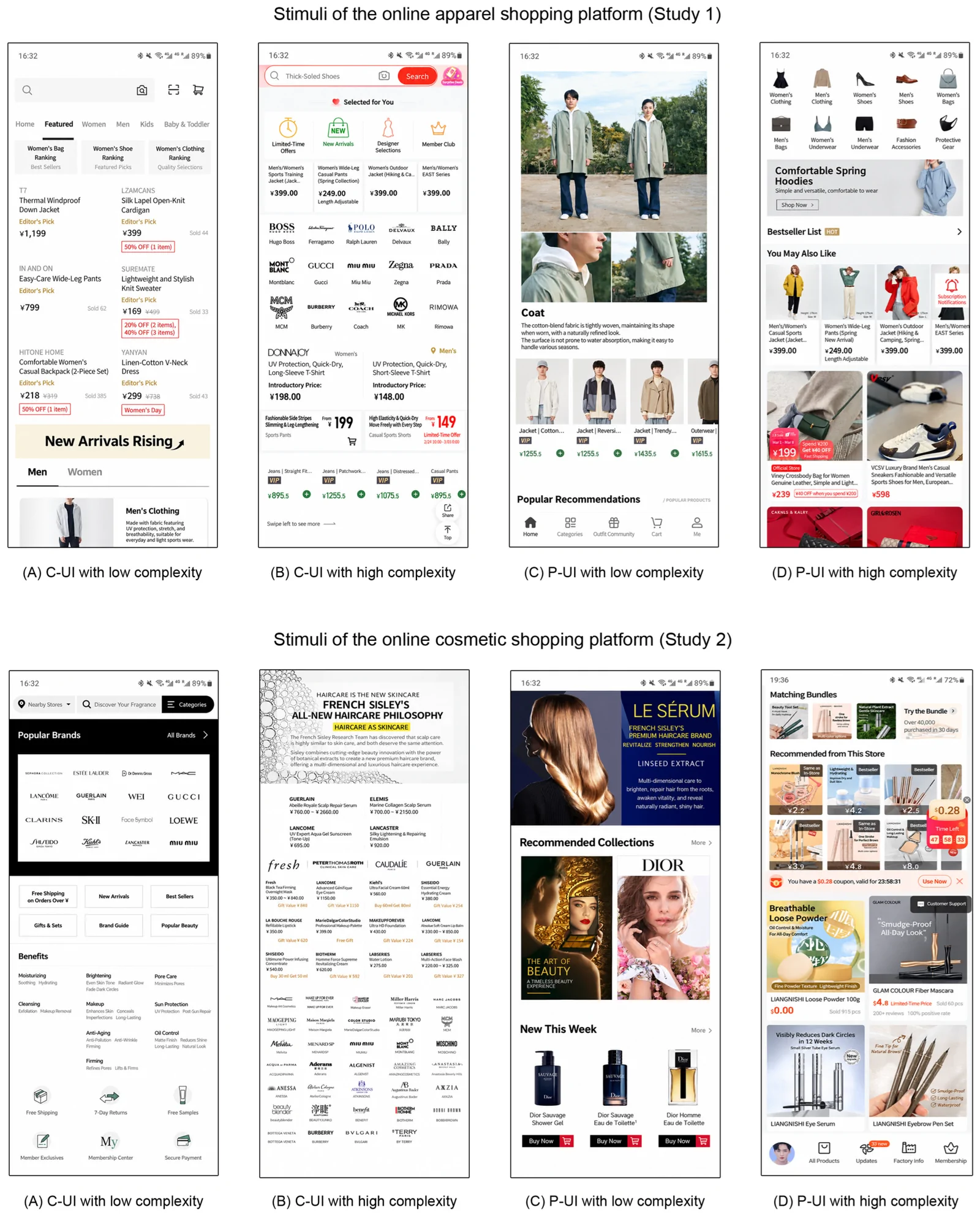
Experimental interface stimuli used in the two studies. The upper row presents the apparel-shopping stimuli used in Study 1, and the lower row presents the cosmetics-shopping stimuli used in Study 2: (**A**) C-UI with low complexity; (**B**) C-UI with high complexity; (**C**) P-UI with low complexity; (**D**) P-UI with high complexity.

## Data Availability

The data supporting the findings of this study are available from the corresponding author upon reasonable request. The data are not publicly available due to privacy and ethical restrictions.

## Appendix A. Measures Used in the Reported Analyses

**Table A1:** Perceived Ease of Use.

Item	Statement
PEOU1	Learning to operate this app is easy for me.
PEOU2	My interaction with this app is clear and understandable.
PEOU3	It is easy for me to become skillful at using this app.

**Table A2:** Perceived Shopping Quality.

Item	Statement
PSQ1	I am confident in the expertise demonstrated by this app; it appears to know what it is doing.
PSQ2	I am satisfied with the services this app provides.
PSQ3	I am satisfied with my overall experience with this app.
PSQ4	I would recommend this app to others.

Note. All items were measured using a seven-point Likert scale ranging from 1 (“strongly disagree”) to 7 (“strongly agree”). Items were adapted from established measures and modified to fit the context of online fashion-shopping applications.

**Table A3:** Coding of Consumer Characteristics.

Variable	Measurement/Coding
Gender	Self-reported categorical variable
Age	Seven age categories, ranging from under 18 years to over 60 years
Years of Online Shopping	Less than 1 year; 2–3 years; 4–5 years; more than 6 years
Shopping Frequency	Once per week or less = 1; twice or more per week = 0

Note. Shopping frequency was dichotomously coded for the moderated moderation analyses.

## Appendix B. Experimental Conditions and Stimulus Design

**Table A4:** Experimental Interface Conditions.

Condition	Interface Design Orientation	UI Complexity	Design Characteristics
A	C-UI	Low	Text- and information-oriented presentation with relatively limited visual stimulation; simplified layout and clearer navigation
B	C-UI	High	Text- and information-oriented presentation with denser information, smaller interface elements, and less intuitive navigation
C	P-UI	Low	Image-rich, visually salient, and interactive presentation combined with a simplified layout and clear navigation
D	P-UI	High	Image-rich, visually salient, and interactive presentation combined with greater information density and a more complex interface structure

Note. C-UI = central-route-oriented user interface; P-UI = peripheral-route-oriented user interface. These labels represent theoretically informed interface design orientations rather than directly observed cognitive processing states. The same 2 × 2 experimental structure was applied in Study 1 (apparel) and Study 2 (cosmetics), with product-specific interface stimuli.
